# *Notes from the Field:* Legionnaires Disease in a U.S. Traveler After Staying in a Private Vacation Rental House in the U.S. Virgin Islands — United States, February 2022

**DOI:** 10.15585/mmwr.mm7220a5

**Published:** 2023-05-19

**Authors:** Valerie V. Mac, Katie Labgold, Heidi L. Moline, Jessica C. Smith, Jamaal Carroll, Nakia Clemmons, Chris Edens, Brett Ellis, Cosme Harrison, Kelley C. Henderson, Maliha K. Ishaq, Natalia A. Kozak-Muiznieks, Jasen Kunz, Marlon Lawrence, Claressa E. Lucas, Heather L. Walker, Melisa J. Willby, Esther M. Ellis

**Affiliations:** ^1^Epidemic Intelligence Service, CDC; ^2^U.S. Virgin Islands Department of Health; ^3^Division of Bacterial Diseases, National Center for Immunization and Respiratory Diseases, CDC; ^4^Division of Environmental Health Science and Practice, National Center for Environmental Health, CDC; ^5^Division of Foodborne, Waterborne, and Environmental Diseases, National Center for Emerging and Zoonotic Infectious Diseases, CDC.

On February 1, 2022, the U.S. Virgin Islands (USVI) Department of Health (VIDOH) was notified of a confirmed case of Legionnaires disease in an adult U.S. resident (Figure). The patient, a man aged 55 years, returned to his U.S. state of residence from leisure travel in USVI on January 22 and developed a cough, shortness of breath, and fatigue on January 23. On January 29, he was hospitalized for shortness of breath and received a positive SARS-CoV-2 test result at admission. The combination of the patient’s symptoms and recent travel history prompted administration of a urinary antigen test (UAT) for Legionnaires disease specific to *Legionella pneumophila* serogroup 1 (Lp1); a positive result was returned on January 31. Inpatient treatment administered for COVID-19 pneumonia and Legionnaires disease included remdesivir, oral levofloxacin, oral and intravenous steroid therapy, and as-needed use of a bronchodilator inhaler and an expectorant. Remdesivir was discontinued during inpatient treatment because of elevated liver enzymes. The patient recovered and was discharged on February 2.

**FIGURE:**
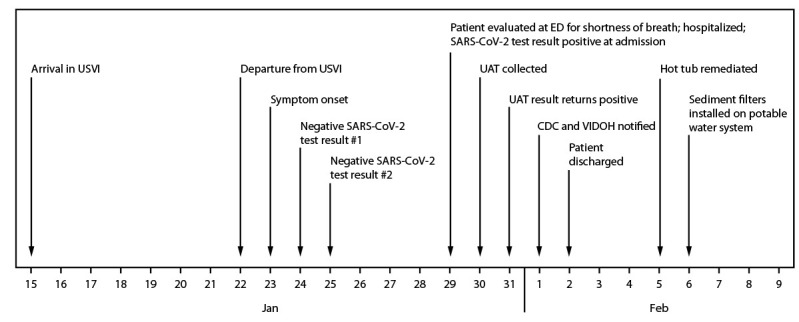
Time line of patient travel, illness onset, diagnosis, and environmental remediation for a case of Legionnaires disease in a U.S. traveler visiting the U.S. Virgin Islands — United States, January–February 2022 **Abbreviations:** ED = emergency department; UAT = urinary antigen test; USVI = U.S. Virgin Islands; VIDOH = Virgin Islands Department of Health.

Interviews with the patient indicated that he had stayed at a privately owned vacation rental property during January 15–22. As is the case with most USVI residential properties, rainwater collected into a cistern under the home was the property’s potable water source (*1*), which supplies water for drinking, bathing, a swimming pool, and two hot tubs. Water is heated by a solar water heater, which does not allow for water temperature control. The property owner reported that no routine chlorine treatment or water filtration systems were used to maintain the potable water source during the patient’s stay.

On February 3, VIDOH requested assistance from CDC’s Legionella program to conduct environmental sampling and testing for *Legionella* bacteria. Twenty-five bulk water, swab, and cartridge filter samples were collected at the property. *L. pneumophila* nonserogroup 1 was detected in 11 locations, including one hot tub cartridge filter, all showers, the two sampled bathroom sinks, and two critical control points: the cistern and solar water heater. Lp1, the only strain detectable by UAT, was not detected in environmental samples.

No respiratory specimen was collected from the patient, which would have been needed to detect and directly link an *L. pneumophila* nonserogroup 1 infection to the property; however, *L. pneumophila* of any serogroup can infect humans, and any environment hospitable to *L. pneumophila* nonserogroup 1 is also hospitable to Lp1 (*2*,*3*). Thus, even without a direct linkage of the *L. pneumophila* strain detected in the patient to the property, the high prevalence (44%) of samples positive for *L. pneumophila* nonserogroup 1 in environmental samples collected at a single time point revealed favorable environmental conditions for widespread, uncontrolled *Legionella* growth of multiple serogroups at the property.

Given the detection of *L. pneumophila* nonserogroup 1 at multiple sampling locations on the same day, VIDOH provided recommendations to disinfect the property’s plumbing system and implement water system maintenance (installing a multistage ultraviolet filtration system and performing routine chlorination). The property owner completed remediation recommendations during February 5–6. However, a request by VIDOH for retesting in September was declined by the property owner, highlighting a gap in VIDOH’s ability to evaluate maintenance effectiveness.

Vacation rental properties represent a growing proportion of the accommodation types identified in U.S. travel-associated Legionnaires disease cases and outbreaks (*4*). In resource-constrained settings such as USVI, commonly recommended water quality maintenance strategies (e.g., controlled temperature water heating and multistage water filtration) are not easily implemented, highlighting territory-specific potable water maintenance and testing needs. In light of these maintenance challenges, and that an estimated 90% of USVI residences rely on cisterns as their potable water source, the environmental assessment and sampling results of this investigation underscore the potential for undetected Legionnaires disease cases among USVI residents and travelers (*1*). Patients with clinical signs consistent with Legionnaires disease such as shortness of breath, cough, fatigue, and a history of travel to USVI should be tested for *Legionella*, even if, as was the case for the patient described in this report, another respiratory virus test result is positive. When cases are identified, environmental assessment and sampling, remediation strategy implementation, and timely postremediation testing are central to ensuring treatment success. VIDOH continues to work with CDC’s *Legionella* program to improve territory Legionnaires disease case surveillance, *Legionella* environmental assessment and sampling practices, and educational outreach to vacation rental owners.
